# Clinical Limitations of Photon, Proton and Carbon Ion Therapy for Pancreatic Cancer

**DOI:** 10.3390/cancers12010163

**Published:** 2020-01-09

**Authors:** Mikaela Dell’Oro, Michala Short, Puthenparampil Wilson, Eva Bezak

**Affiliations:** 1 Cancer Research Institute and School of Health Sciences, University of South Australia, Adelaide SA 5001, Australia; michala.short@unisa.edu.au (M.S.); eva.bezak@unisa.edu.au (E.B.); 2 Department of Radiation Oncology, Royal Adelaide Hospital, Adelaide SA 5000, Australia; puthenparampil.wilson@unisa.edu.au; 3 School of Engineering, University of South Australia, Adelaide SA 5001, Australia; 4 Department of Physics, University of Adelaide, Adelaide SA 5005, Australia

**Keywords:** pancreatic cancer, proton therapy, carbon ion therapy, stereotactic body radiation therapy, hypoxia activated prodrug, radiosensitizer

## Abstract

Introduction: Despite improvements in radiation therapy, chemotherapy and surgical procedures over the last 30 years, pancreatic cancer 5-year survival rate remains at 9%. Reduced stroma permeability and heterogeneous blood supply to the tumour prevent chemoradiation from making a meaningful impact on overall survival. Hypoxia-activated prodrugs are the latest strategy to reintroduce oxygenation to radioresistant cells harbouring in pancreatic cancer. This paper reviews the current status of photon and particle radiation therapy for pancreatic cancer in combination with systemic therapies and hypoxia activators. Methods: The current effectiveness of management of pancreatic cancer was systematically evaluated from MEDLINE^®^ database search in April 2019. Results: Limited published data suggest pancreatic cancer patients undergoing carbon ion therapy and proton therapy achieve a comparable median survival time (25.1 months and 25.6 months, respectively) and 1-year overall survival rate (84% and 77.8%). Inconsistencies in methodology, recording parameters and protocols have prevented the safety and technical aspects of particle therapy to be fully defined yet. Conclusion: There is an increasing requirement to tackle unmet clinical demands of pancreatic cancer, particularly the lack of synergistic therapies in the advancing space of radiation oncology.

## 1. Introduction 

Pancreatic cancer is the seventh most lethal solid tumour worldwide. Independent of the disease stage, the 5-year survival rate remains at 9% [1]. Optimal treatment typically involves a multimodality approach of surgical resection combined with chemotherapy and/or radiation therapy [2]. The local control (LC) and overall survival (OS) rates remain low for these patients despite 40–50% presenting metastasis-free locally advanced pancreatic cancer (LAPC) [3]. Currently, complete resection provides the only cure with uncertainty surrounding pre- or post-surgical chemoradiation [4].

Current therapeutic approaches are limited by hypoxia and blood barrier-like reductions in efficiency of pre- or post-surgical chemoradiation. Solid LAPC tumours with hypoxic cells not only reduce the effectiveness of photon radiation therapy (XRT) but also limit the intake of chemotherapeutic agents. Conventionally, 3D conformal radiation therapy (3DCRT) has previously proven insufficient in terms of achieving satisfactory tumour control probability (TCP) and normal tissue complication probability (NTCP) [5]. Modulated XRT techniques such as intensity modulated radiation therapy (IMRT) and volume modulated arc radiation therapy (VMAT) are now well established standards of care at reducing NTCP in clinical practice. However, the role of XRT for LAPC remains controversial as chemoradiation has not proven to significantly impact TCP for LAPC patients, irrespective of the technique used [6,7,8,9].

Recent advancements in delivery, image guidance and planning have guided novel techniques of radiation dose escalation. Stereotactic body radiation therapy (SBRT) allows larger doses of up to 25 Gy per fraction to be delivered (as opposed to 2 Gy per fraction), increasing the radiobiological effectiveness of XRT. However, due to the anatomical location of the pancreas and proximity to critical normal structures, doses required to provide sufficient LC are still prohibitive [10].

**Figure 1:**
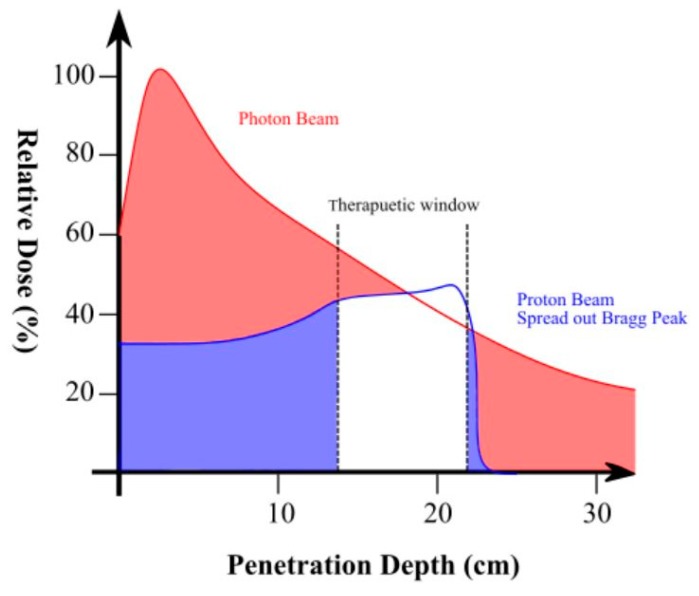
Spread out Bragg Peak of proton therapy (red) compared to photon radiation therapy (blue). The shaded areas represent relative dose to normal tissue.

Advancements in particle therapy (e.g., proton and carbon ion) provide a different solution to delivering enhanced biological damage compared to XRT due to more advantageous dose deposition based on the Bragg Peak (Figure 1) [11]. The physical properties of charged particle dose deposition in tissue result in sharper dose distribution enabling tolerable full-dose radiation delivery whilst minimising NTCP to surrounding organs-at-risk (OAR). Therapeutic resistance in the tumour microenvironment, however, may remain as the relative biological effectiveness (RBE) of proton therapy (PT) is only 10% higher than XRT [12]. As a result, investigations into heavier particles which induce more severe DNA damage continue (compared to both PT and XRT). Carbon-ion therapy (C-ion) utilises charge carbon-12 ions of larger mass to increase direct DNA damage effectiveness by a factor of approximately two to four [13,14,15,16]. Recently established at a handful of institutions, the improved dose conformation and potential for overcoming effects of hypoxia is currently being investigated as a potential solution for LAPC [11,17].

Alternate pathways to combat LAPC radiation resistance are also being investigated, including the implementation of synergistic agents. Hypoxia activated drugs (HAPs) are synergistic agents introduced to overcome this longstanding radiobiological challenge of hypoxia. The enhanced anti-tumour activity of HAPs combined with conventional and new chemoradiation treatments has long been hypothesised across several cancer sites; however, the clinical implementation has been vastly under-explored [18].

The aim of this work was to review the current status of combined knowledge for XRT, PT and C-ion therapy. This paper will look at the reported results of chemoradiation trials using PT, C-ion and XRT to determine if either modality has impacted OS, with the emphasis on radiation modality only. Similarly, the current pre-clinical and clinical status of hypoxia-targeted treatment combined with or without XRT will be assessed for effectiveness in the treatment of pancreatic cancer.

## 2. Methods

A search strategy was conducted using MEDLINE^®^ database in April 2019 with the relevant key terms encompassing the themes of: proton therapy, pancreatic cancer and hypoxia targeting adjuvant therapy (Appendix A). Limitations were applied to the search, including: English language, published from 2000 to present. The search identified 87 papers. Conference abstracts, duplicates across the searches and papers which involved 3DCRT treatments were excluded (as per the exclusion criteria in Appendix B). A total of 38 papers were exported for the purpose of this review. The reference lists were pearled for other literature (including C-ion trials), resulting in 82 papers in total.

This systematic review was performed according to the PRISMA statement (http://www.prisma-statement.org/). All methods for exclusion criteria, data extraction and quality assessment were specified in advance. However, due to the evaluated number of participants, variation in reporting and data collection a systematic analysis was not possible due to the lack of consistency. Data were extracted and tabulated to provide a clear integrative overview of the results to date for chemoradiation for pancreatic cancer. The review protocol was not registered with any organisation.

Most the trials discussed have used chemotherapy, however, we have not compared the individual chemotherapy schedules. The chemoradiation combinations are tabulated and where appropriate, we commented on the side effects of the chemoradiation.

## 3. Results and Discussion

### 3.1. Combating Hypoxia in Pancreatic Cancer

Historically, some of the largest prospective phase III chemoradiation trials for pancreatic cancer, including the European Study Group for Pancreatic Cancer-1 (ESPAC-1) and RTOG 97-04 trials, found that neither chemotherapy nor XRT alone could provide tumour control [19,20]. The results extended into single- and multi-agent gemcitabine chemoradiation trials such as LAP07, showing no statistical impact on OS [8]. Despite these trials utilising 3DCRT, now inadequate compared to IMRT and VMAT, they all demonstrated the radiation resistance of pancreatic cancer. Hypoxia has long been recognised as the main cause of radiation resistance in pancreatic cancer [21]. Failure of studies to account for the presence of hypoxia has prevented them reaching meaningful survival endpoints [22]. As such, there is a clinical need to address hypoxia in pancreatic cancer and develop novel therapies that specifically target and exploit these oxygen-deficient regions in order to improve systemic and local therapies. Oxygenating these hypoxic regions can affect the lethality of photon radiation therapy by threefold [23].

To date, even the most clinically effective chemoradiation regimen had little impact on TCP due to the reduced permeability of the stroma and heterogeneous blood supply to the tumour [24]. Several strategies have demonstrated limited clinical benefit at reintroducing oxygen to regions of the tumour which harbour hypoxic cells prior to XRT. Some strategies include hyperbaric chambers, high oxygen-content gas breathing, invasive needle insertions, and blood transfusions [25].

Hypoxia targeting drugs have been developed to synergistically enhance fractionated XRT by targeting a variation of physiological characteristics and molecular pathways of tumours [18]. Of particular interest are the successful clinical trials investigating the concept of hypoxia-activated prodrugs (HAPs); a bioreductive agent which is metabolised into an active vasodilating drug in hypoxic tumour tissue [26].

### 3.2. Hypoxia Activated Prodrug

Extensive preclinical evaluations of Evofosfamide (TH-302) Threshold Pharmaceuticals (South San Francisco, CA, USA) have demonstrated promising antineoplastic properties in the treatment of pancreatic cancer, improving the clinical efficacy of chemotherapy and/or XRT [18,27,28]. TH-302 is a prevalent “dual-function” radiosensitising agent: producing a potent DNA-alkylating species which damage DNA in both proliferating and quiescent cells and modify free radical damage [23,29]. TH-302 unique biochemical properties allow it to be relatively non-toxic to normoxic tissues through activation of cytotoxic products in hypoxic conditions only [23].

The possibility of TH-302 working concurrently with chemoradiation for pancreatic cancer have long been tested in pre-clinical and in vitro studies [23,30,31]. The addition of TH-302 improved the outcome of chemoradiation for pancreatic xenograft models, overcoming the biological impact of hypoxia. Lohse, et al. [23] combined fractionated XRT with TH-302 to significantly delay growth and reduce tumour volume in patient-derived xenograft models; a result seen in neither XRT or TH-302 treatments alone. A result was more dominant in rapidly growing patient-derived pancreatic xenograft models compared to slow growing hypoxic models. This impacts on tumour growth rates, which is a strong predictor of OS for clinical application and efficacy of the treatment combination.

Borad, et al. [27] conducted the first randomised phase II clinical trial to demonstrate the potential outcomes of combining TH-302 with gemcitabine alone. The dual-drug combination extended to the global placebo-controlled randomised phase III “MAESTRO” trial (NCT01746979) with contrasting results. The results demonstrated no OS benefit from combining TH-302 with gemcitabine (median of 8.7 months compared to 7.6 months for gemcitabine alone) [32]. However, the treatment combination demonstrated favourable signs of antitumour activity regarding patient PFS (median of 5.5 months compared to 3.7 months for gemcitabine alone) and higher objective response rate [33]. Ideally, TH-302 and gemcitabine should not be used alone but in combination with XRT to enhance the biological damage. These contradictory results leave a gap for further research examining TH-302 combined with chemoradiation for pancreatic cancer.

The use of TH-302 with chemotherapy was ineffective at providing a statistically significant impact on patient outcome; however, as previously mentioned, the combination with XRT in vivo and in vitro studies showed potential. The first in-human clinical trial testing TH-302 with chemoradiation was planned to be a phase I non-randomised, single-arm, trial in Dutch oesophageal adenocarcinoma patients (NCT02598687) [34]. Although not pancreatic, the similar anatomical location of the oesophageal adenocarcinoma (at the oesophago-gastric junction) had potential to give insight demonstrating the NTCP effects of the trimodality therapy on radiosensitive gastric OAR. However, due to the previous failure of TH-302 to reach its primary endpoint when combined with chemotherapy alone in trials for soft tissue sarcoma (NCT01440088) and pancreatic cancer (MAESTRO), the study was eventually withdrawn [33,35].

### 3.3. HAPs Limitations and Future Work 

Major restrictions in HAPs development are from functional introduction by the unpredictable tumour vasculature which prevents regular implementation [25]. Dose dependence and fibrosis at the site of prolonged injection were limitations experienced during TH-302 testing. Other classes of drugs have been limited by functional requirements such as local administration, speed of breakdown and tolerability [18]. Investigations continue into alternate HAPs in the clinical context of pancreatic cancer, see Table 1 for an overview of past and current studies.

### 3.4. Stereotactic Body Radiation Therapy

The introduction of advanced XRT techniques and procedures such as breath-hold, real-time tumour tracking, respiratory motion reconstructions and soft tissue matching have allowed dose escalation to be safely administered. SBRT further refines the target conformality of XRT to feasibly deliver a higher Biologically Effective Dose (BED) in a shorter period in order to improve LC for LAPC patients from 79% to 94%, translating to an increased OS [3,36,37,38,39,40]. The SBRT chemoradiation trial results summarised in Table 2 are difficult to compare and interpret between studies due to the diversity of fractionation schemes, recording of statistics and inhomogeneity of recruited patients (e.g., respectability and disease status).

Single-fraction SBRT was originally investigated as advantageous based on convenience, reduction in interference of systemic therapy and intensification of dose in order to improve TCP. Chang et al. [22] is the largest retrospective study of the published single-fraction SBRT trials (including 40 participants from previous studies by Schellenberg et al. [41], Koong et al. [42] and Koong et al. [38]. Single-fraction SBRT for unresectable pancreatic cancer patients at Stanford University demonstrated promising LC rates between 75% and 100% but relatively unaffected MST (11–11.8 months). However, this large reported series was limited by a shorter median follow up time (5–9.1 months). Additionally, the inability for a single-fraction to exploit the reoxygenation of hypoxic tumour cells could have also potentially prevented the treatment modality from making a meaningful impact on survival rates.

Pollom, et al. [43] retrospectively analysed the outcomes of 167 patients who underwent either single- vs multi-fraction SBRT in a bid to determine the optimal radiation treatment schedule. Minimal difference existed for the survival rates between the single- and multi-fraction groups, with no compromise on LC. There were, however, significantly fewer acute grade > 2 and late grade ≥ 3 GI toxicities in multi-fraction SBRT, despite the multi-fraction group having a larger median PTV.

Acute GI toxicity for SBRT is not substantially different from that of conventionally fractionated XRT; however, the incidence of late toxicity remains a concern especially in the context of pre-surgical downstaging. Combined with concurrent chemotherapy several SBRT studies experienced a trade-off of LC for late grade ≥ 2 GI toxicities [44]. Though the quick course is favourable for LAPC clinical management, surgery is still the only curative treatment [5,45]. SBRT studies, by Mellon, et al. [46] and Chuong et al. [37] demonstrated 51% and 56% of borderline resectable patients, respectively, were able to undergo post-SBRT resection with high complete resection rates. Mellon et al.’s downstaging of initially unresectable disease successfully resulted in an increased median OS (14.0 months for unresected patients vs. 34.2 months for surgically resected patients) [46]. In addition, Chuong et al. [37] demonstrated that a further 10% of LAPC patients underwent post-SBRT resection with curative intent.

Previously, larger target margins (median planning target volume of 136 cm^3^) encompassed more of the duodenal mucosa and increased number of fields resulted in higher incidence of late grade ≥ 2 GI toxicities (up to 94% in Hoyer, et al.’s [47] multi-fraction SBRT study) [41]. More recently Chuong, et al.’s [37] and Comito, et al.’s [40] fractionated SBRT studies demonstrated a lower incidence of acute toxicities (grade 3 ≥ 0) for median planning target volumes of (111.01 and 64.7 cm3, respectively) paired with a high 1-year LC (≥ 81%) and MST (15 and 19 months, respectively) for LAPC patients. 

Even with advancements in precision-guided protocols of XRT current limitations still exist in radiation resistance of the tumour microenvironment. SBRT remains controversial due to the decreased time for tumour reoxygenation [23,48,49]. In addition to reducing interruptions to systemic therapy and improving patient quality of life, the clinical impact of SBRT still remains modest at best. Therefore, future studies are required to better integrate systemic therapy with SBRT in order to improve OS whilst lowering integral dose to OAR. A gap which may possibly be exploited by particle therapy and the further development of systemic therapies such as HAPs.

### 3.5. Proton Therapy

Summary of PT clinical studies in Table 3 demonstrates an improved 2-year OS and MST ranging from 31–50.8% and 18.4–25.6 months [45,50]. The enhanced biological damage of PT radiation dose delivery compared to XRT (i.e., dose reporting) across the studies varies in terms of: RBE, cobalt Gray equivalents (CGE refers to absorbed dose × 1.1 (RBE) to express the biologic effective proton dose), Gray equivalents (GyE refers to proton physical dose (in Gray) × 1.1 (RBE)). We have used the individual publications’ definitions in our Table 3 of PT chemoradiation studies (Table 3).

Several current PT studies compare their work to 3DCRT trial data which delivered a significantly higher integral dose than newer treatment modalities. The rate of upper GI toxicity was compared with 3DCRT studies which inherently use larger radiation field sizes. Studies as late as 2012 still compared dose to the irradiated small bowel and side effects from PT with patients undergoing 3DCRT, demonstrating a gap between conventional XRT and particle therapy [51]. As such further studies are required to compare the latest techniques (IMRT) in order to compare the clinical efficiency of current particle therapy treatment.

Studies previously linked aggressive radiation therapy dose (67.5–70.2 GyE), concomitant delivery of full-dose gemcitabine and size and/or orientation of radiation therapy fields (inclusion of prophylactic nodal regions) to an increase in high grade GI toxicities. As seen in the Table 3, incidence of grade 3 GI toxicity largely varies across PT studies from 0% and 50%.

First attempts at dose escalation performed by Terashima, et al. [52] and Takatori, et al. [53] observed a particularly high incidence (50%) of GI radiation-induced ulcers of 50 and 126 enrolled patients, respectively. The two studies from Hyogo Ion Beam Medical Center employed a significantly a higher prescription of 2.5 to 2.7 GyE per fraction compared to other PT studies in Table 3. Late grade ≥ 3 GI effects (10%) incidence was significantly higher than SBRT such as Mahadevan, et al.’s [54] and Herman, et al.’s [55] SBRT studies (6%). Speculation surrounded contributing factors such as radiation field size (including regional lymph nodes) and orientation increased the rate of upper GI toxicities [56]. These factors combined with the higher proton RBE reported for the distal end of the Bragg peak may have also influenced higher occurrence rates of late Grade 3 GI toxicities [12].

Ongoing developments in PT have demonstrated favourable dosimetry in order to facilitate hypofractionation and dose escalation. Kim, et al.’s [57] and Jethwa, et al.’s [58] recent retrospective studies observed no late or acute grade ≥ 3 GI toxicities. Kim, et al.’s [57] multivariate analysis identified that induction chemotherapy was a significant factor for overall survival (21.6 months vs. 16.7 months). MST remained mildly improved (19.3 months) for patients with localized inoperable disease until Hiroshima et al.’s latest PT study. Hiroshima, et al. [45] finessed higher dose delivery (50 GyE with a 17.5 GyE boost) through opposed anterior-posterior beam arrangement reporting an increased MST of 25.6 months and 1-year OS of 77.8%. Altering Terashima et al.’s additional boost field by 10% of the dose prescription, Hiroshima, et al. [45] demonstrated no grade ≥ 2 GI acute or grade ≥ 3 GI late adverse events. 17 patients who received the concomitant boost up to 67.5 GyE demonstrated an improved median OS of 42.5 months and median time to local recurrence of >36 months. Concluding a PT dose of up to 67.5 GyE predicts a significant improvement in OS and LC of pancreatic cancer patients.

To date, while PT clinical results have had a positive impact on MST and OS for pancreatic cancer treatment, no chemoradiation combination has resulted in a statistically significant increase in survival. Investigations into concomitant capecitabine by Sachsman et al. [50] and Nichols, et al.’s [59] reported no acute grade 3 GI toxicities and minimal acute grade 2 GI toxicities (9% and 13.6%, respectively). All 3 patients who received grade 2 acute GI toxicities in Nichols, et al. [59] prospective PT trial received anterior and lateral beams. Based on evidence that a more heavily weighted posterior-anterior field eliminated grade 2 GI toxicity and improved medians weight lost, this arrangement was then modified for the successive 19 patients.

Concern regarding the increased risk of surgical complications and late effects of combined therapy on the GI tract tissue prior to treatment have guided investigations into post-operative PT. Suggesting that post-operative PT may fail due to the extended time period required for the surgery to heal, Hitchcock et al.’s [60] pre-operative study demonstrated five initially unresectable patients becoming resectable and resultant improved median OS of 24 months (range, 10–30).

Pre-operative chemoradiation reduces resource (and cost) demand and required appointments for a patient to attend compared to long fractionation schedules. Tolerability of short course pre-operative radiation therapy has proven feasible in combination with capecitabine [61]. In particular, PT studies by Hong, et al. [62], Hong, et al. [63] and Tseng, et al. [64] demonstrated improved surgical resection and tolerability (acute grade ≥ 3 GI toxicity ≤ 27%).

PT treatment schemes for LAPC patients currently being investigated include the feasibility proton reirradiation after SBRT, combination therapy or simultaneous integrated boost in PT [65]. Reviews have emphasised the requirement for multimodality treatment exploration in controlled clinical trials in order to make a meaningful impact on LAPC outcome [4]. However, this does not fix the fundamental issue of hypoxia and/or improve the outcomes of OS. More investigations into the effectiveness and long-term outcomes of PT for LAPC are therefore required. Pairing dose escalation and concomitant boost technique and the physical advantages of PT with improved systemic drugs could further improve treatment outcomes.

### 3.6. Carbon Ion

At the time of this review, only four C-ion chemoradiation studies had published the outcomes for LAPC patients, all from Japan. A summary of these studies along with published recruiting and withdrawn studies are in Table 4.

The National Institute of Radiological Sciences, is responsible for most of the published particle therapy reports in pancreatic cancer patients, demonstrating higher LC using the heavy C-ion therapy. Despite these promising preliminary results, the use of C-ion for these radioresistant tumours is vastly under-explored; contributing to 5.4% of their workload [66]. C-ion studies available for comparison (Table 4) demonstrate similar survival rates across Table 2 and Table 3 in terms of median OS, 1- and 2-year FFLP/LC. However, C-ion provided similarly high rates of GI toxicities as PT.

As demonstrated in Table 4, current C-ion studies successfully delivered up to 55.2 GyE in 12 fractions. Shinoto, et al. [67] performed the first observational trial to combine full-dose gemcitabine with an escalated C-ion dose (55.2 GyE) demonstrating similar survival (MST of 19.6 months and 2-year FFLP of 83%) to Hiroshima et al.’s PT study (MST of 25.6 months and 2-year LC of 78.9%) for LAPC patients. Current recommendations for C-ion LAPC treatment are therefore full-dose gemcitabine (1000 mg/m^2^) with 55.2 GyE in 12 fractions.

Shinoto, et al. [68], more recently, validated the efficacy and safety of 55.2 GyE, demonstrating an increase in OS rates (2-year OS rose from 48% in 2016 to 53% in 2018) with acceptable late (3%) and acute (0%) grade 3 GI toxicities. The maximum tolerated dose was evaluated as safe, under the conditions of respiratory-gating and stringent selection criteria. Although the enrolled participants demonstrated a range of target volume to GI tract distances (range: 0 mm to ≥ 10 mm) not all patients were selected for C-ion therapy. Patient eligibility for C-ion remains based on the relationship between tumour to GI tract distance and achievability of dose constraints, potentially skewing the incidence and severity of GI toxicities as only patients with favourable GI tract distances were selected for these trials.

### 3.7. Gaps in Particle Therapy for Pancreatic Cancer

Similar complications are of concern with C-ion as with PT, including radiation-induced ulcers in the stomach and duodenum and intraoperative fibrosis. Fukumitsu, et al.’s [65] PT simulation study correlated an increase in GI toxicities to proximity of target volume to GI tract, causing it to receive increased high radiation dose. Kawashiro, et al. [69] tested the feasibility of C-ion finding it non-ideal for tumours located in close proximity (≥ 5 mm) to the GI tract (unrelated to the anatomical location within the pancreas head, body or tail). 

Another contributing factor to increased radiation-induced ulcers is the determination/uncertainties of RBE and biological optimization using planning algorithms for PT and C-ion. RBE for particle therapy is a complex function and estimated planned dose may vary when clinically translated, contributing to unforeseen acute and/or late toxicities. Feasibility and tolerability of the physical properties of C-ion chemoradiation such as a higher concentration of particles and concern of fibrosis in a pre-operative setting was initially tested by Shinoto, et al. [70]. A 5-year survival rate of 52% was estimated for the 21 patients who underwent surgical resection. The reduction in penumbra of C-ion reduced the damage to surrounding normal tissue, resulting in minimal change to the tissue during surgical resection and negligible fibrosis (with a similar time delay between RT and surgery between both studies).

As previously discussed in PT; the high rates of toxicity have been attributed to several confounding factors including aggressive radiation therapy dose (67.5–70.2 GyE), high dose per fraction (2.7 GyE), concomitant delivery of ≥800 mg/m^2^ gemcitabine and size and/or orientation of radiation therapy fields (inclusion of prophylactic nodal regions). 

Shinoto, et al.’s [70] C-ion study resulted in patterns of initial disease progression (65% of the patients experiencing distant metastasis and 8% regional recurrence) in the absence of chemotherapy. Established as a broad ranging anti-tumour treatment gemcitabine is the most widely recommended chemotherapy agent to reduce this risk of distant metastasis and regional recurrence, the major mode of LAPC treatment failure. However, administration of gemcitabine has been linked to a higher incidence of grade ≥ 3 haematological toxicities across chemoradiation trials (Table 2, Table 3 and Table 4). Maemura, et al.’s [71] comparative PT study of 25 patients (10 undergoing PT and 15 undergoing hyper-fractionated XRT) only had two patients develop grade ≥ 2 gastric ulcers, still appearing advantageous compared to XRT and C-ion regarding high grade haematological toxicity when gemcitabine was employed. In fact, Hiroshima, et al.’s [45] PT trial reported all grade ≥ 3 and 4 events were haematologic and correlated with full-dose gemcitabine and/or speculated as high doses to the spleen (as previously described in XRT studies [72,73]). 

**Figure 2:**
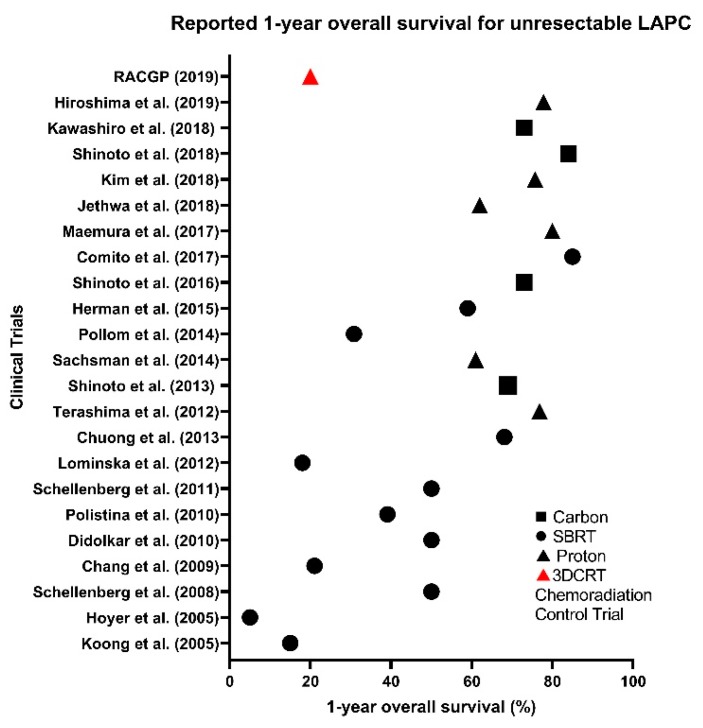
Reported 1-year overall survival (OS) rate for unresectable LAPC across SBRT (●), proton (▲) and carbon (∎) clinical trials compared to 3D conformal chemoradiation (▲) control trial.

**Figure 3:**
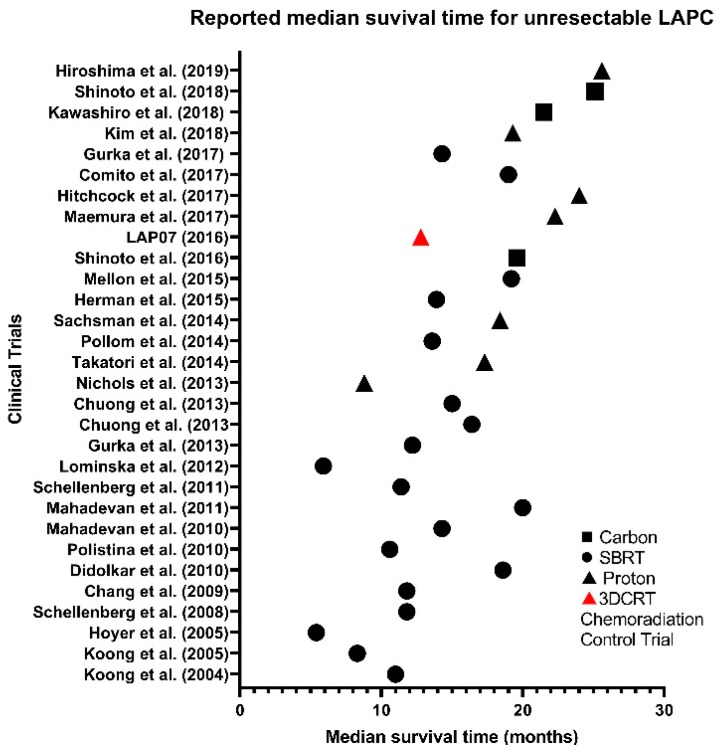
Reported median survival time (MST) for unresectable LAPC across SBRT (●), proton (▲) and carbon (∎) clinical trials compared to 3D conformal chemoradiation (▲) control trial.

**Figure 4:**
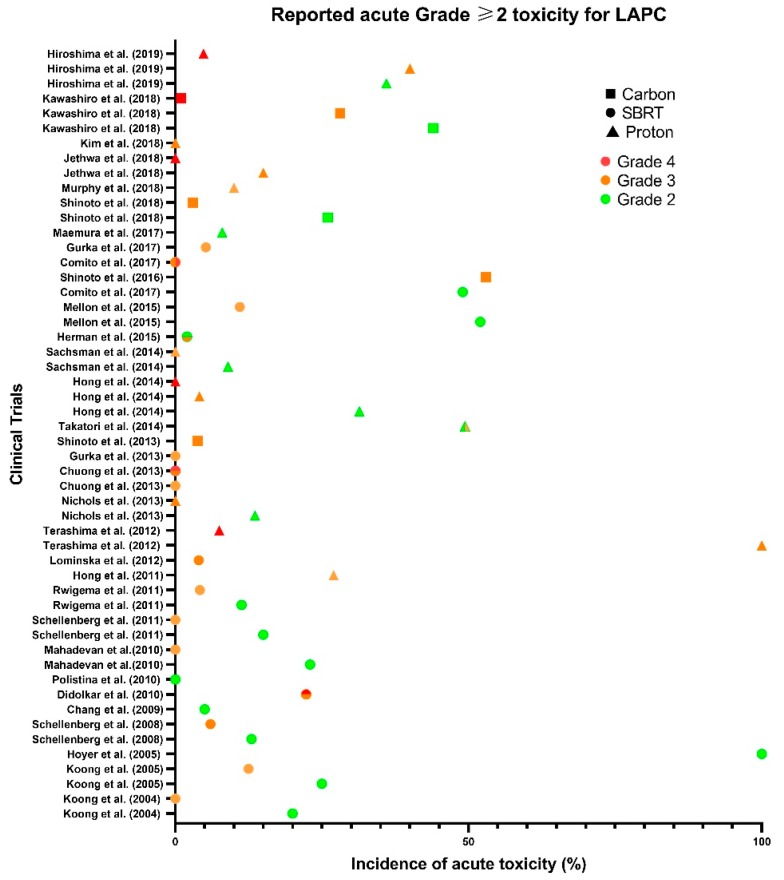
Reported acute Grade ≥ 2 toxicity for resectable and unresectable LAPC across SBRT (●), proton (▲) and carbon (∎) clinical trials. Prescribed dose and fractionation varied across studies.

Developments in particle therapy have marginally improved MST, LC and OS in recent years, whilst marginally reducing the incidence and grade of GI toxicities compared to XRT (Figure 2 and Figure 3). However, high-grade haematological toxicities experienced seem to be irrespective of modality type, and with a requirement for effective systematic therapies to prevent metastases, there seems to be no solution yet (Figure 4) [17,67]. As many patients in Table 4 experienced multiple toxicities, it is difficult to interpret the individual and compounded impact of C-ion chemoradiation.

In the context of tolerability, 53% of Shinoto, et al.’s [67] C-ion patients experienced grade ≥ 3 acute haematologic toxicities, which related to the type and dose of gemcitabine as learnt in Terashima, et al.’s [52] and Takatori, et al.’s [53] PT studies. Seven of Shinoto, et al.’s [67] 11 enrolled patients treated with 55.2 GyE developed grade 1 or 2 ulcers (and 1 patient a grade 3) which may be due to the exclusion of patients who had direct invasion of tumour into the mucosal GI tract surface reducing GI toxicities, as the treatment field was not in close proximity as it was in PT. The retrospective nature of this study, therefore, had the potential for selection bias; patients could be excluded if not consecutively enrolled. The lower rate of grade 3 ulcers in Kawashiro, et al.’s [17] and Shinoto, et al.’s [68] C-ion studies could also be attributed to selection bias of patients as candidates were selected depending on the lesions contact with the GI tract. Selection bias of these results in Table 4 is difficult to translate to a wider population; improved eligibility criteria is therefore required for C-ion studies.

### 3.8. Limitations and Future Work

Throughout this review, several recurring methodological issues were present, which reduces the ability to directly compare and deduce the current status of studies across Table 2, Table 3 and Table 4. Limited details existed in the reporting guidelines of studies such as the date from which OS was measured and the Common Terminology Criteria for Adverse Events used (if there was one recorded), hindering their reproducibility. Studies in this review measure OS statistics either from diagnosis or from the start date of treatment (or both), which reduces the ability to compare the results and, if incorrectly interpreted by researchers, significantly affect expectations of clinical trials. The Common Toxicity Criteria ranged across studies; which may have resulted in different grading of adverse effects. 

Optimal therapeutic strategies need to be investigated now that PT has been confirmed as safe and effective. PT studies focussed on escalating dose whilst minimising grade 3 GI side effects (which hinder surgical resection in pre-operative setting and reduce quality of life). Many studies were difficult to directly compare as the methodology, dose, timing and type of chemotherapy, RT technique, patient position, contrast, daily filling of stomach, beam delivery technique (scattering or scanning) and disease were vastly different. 

The experience with heavy particles is limited to a few institutions, and no conclusion can yet be drawn about their effectiveness or toxicity [74]. Institutions have determined the efficacy of particle therapy compared to XRT, with several prospective and retrospective studies indicating an improvement in outcomes using PT for LAPC. It is difficult to conclude in lieu of randomized control studies with only eight groups having published preliminary clinical data on the treatment of pancreatic cancer patients with PT. Furthermore, PT is often prescribed on the basis of insurance coverage and insufficient evidence and lack of cost-benefit effectiveness to support the funding of PT clinical trials have left a gap in our understanding of the role of PT in LAPC [58]. Potentially pancreatic cancer patients without access to PT could be enrolled in multi-national trials to overcome the ethical equipoise of prescribing XRT modality.

As evident in Table 3, the clinical progress of combined particle therapy is vastly underexplored, and further studies are necessary to obtain more robust data on its effectiveness and toxicity. There needs to be a more detailed examination of the relationship between irradiation dose and outcome [45]. Patients often experienced more than one toxicity throughout their course of treatment (e.g., haematological and GI). Not only are toxicities often recorded only by incidence of grade, they are often not distinguished by type (haematological or GI). It is increasingly hard to decipher the impact of radiation versus systemic therapy, resulting in gaps of knowledge for patient outcomes. More attention needs to be placed on the systemic and consolidative therapies now that the effectiveness of particle therapy has improved, which could reduce the haematological toxicities. Additionally, QUANTEC data [75] is currently based on dose-volume analyses of rectal and cervical cancer patients [72,76]. Finally, there is an increasing requirement to evaluate and estimate the true RBE value of particle therapy treatment.

Inconsistencies in methodology, recording parameters and guidelines have prevented the safety and technical aspects of particle therapy to be fully defined. Investigations are beginning to perform longer follow up times and employ more transparent enrolment criteria (i.e., image staging of LAPC). As evident in this evaluation further research is required worldwide in reporting guidelines.

Future trials should therefore focus on alternate systemic therapies which reduces the risk of distant failures whilst minimising toxicity when combined with particle therapy. Before pairing them with rapidly advancing targeted therapeutic agents, more stringent consistency in reporting is required to deduce the accumulative effect of systemic therapies.

## 4. Conclusions

There is a clear requirement for aggressive multimodality therapy to tackle the unmet clinical demands of pancreatic cancer. Particle therapy is clearly associated with better LC; however, translating this into improved OS will require ongoing investigations into systemic therapies. Phase II trials are required to prospectively validate the results presented in this review. For comparable conclusions to be drawn these international trials would require a consensus on the prescription and reporting guidelines.

Improvements in imaging and medical biomarkers have recently allowed us to identify hypoxic regions. Despite this, a limited number of studies address the biological and clinical challenges of pancreatic cancer suggestive of why attempts at current RT have proven unremarkable. More research and clinical investigations are required which consider the tumour biology and systemic combined therapy to improve patient survival and NTCP. This paper demonstrates that the targeting of hypoxic regions within pancreatic tumours using HAPs compliments already established chemoradiation regimens. Particle therapy still requires improvements in systemic therapy as minor progress has been noted in chemoradiation alone, even with advancing modalities and techniques.

## Figures and Tables

**Table 1:** Overview of studies investigating hypoxia-activated drugs (pHAPs) in pancreatic cancer (2000-Current).

HAP	Author	Chemotherapy	XRT	Study	Sample Size	Limitations	Summary of Applicable Findings
**TH-302**	Weiss, et al. [28]	No	No	Phase I clinical trial	57	Only 2 pancreatic patients in the study	TH-302 was well tolerated during the first phases of monotherapy investigations with only mild concern regarding high-grade skin and mucosal toxicities above 240 mg/m^2^.
	Borad, et al. [77]	Yes	No	Phase I/II clinical study	46	No full publication of results. No XRT	Overall response rate of 21% and median PFS time of 5.9 months across advanced pancreatic cancer patients.
	Sun, et al. [78]	No	No	In vitro		Monotherapy study	TH-302 antitumour activity was reported as dose-dependent.
	Meng, et al. [79]	No	No	In vivo		Monotherapy study	TH-302 requires more severe hypoxia for to produce higher rates of anti-tumour activity.
	Borad, et al. [27]	Yes	No	Phase II clinical trial (NCT01144455)	229	No XRT	First randomised Phase II clinical trial to demonstrate the potential outcomes of combining TH-302 with gemcitabine. Demonstrated improved tumour response and PFS (median 5.6 vs 3.6 months) compared to gemcitabine alone.
	Sun, et al. [80]	Yes	No	In vitro		No XRT	TH-302, gemcitabine and nab-paclitaxel were assessed as tolerable and providing favourable anti-tumour activity.
	Wojtkowiak, et al. [81]	No	No	In vitro and in vivo		Monotherapy study	Identified biomarkers which may predict a significant decrease in tumour growth with TH-302.
	Lohse, et al. [23]	No	Yes	In vitro		No chemotherapy	Reduced tumour growth rates demonstrate a strong predictor of OS for clinical application and efficacy of the treatment combination.
	Van Cutsem, et al. [33]	Yes	No	Phase III ‘MAESTRO’ clinical trial (NCT01746979)	693	No full publication of results. No overall survival benefit with the treatment combination of TH-302 with gemcitabine (median of 8.7 months compared to 7.6 months for gemcitabine alone)	Treatment combination demonstrated favourable signs of antitumour activity regarding patient PFS (median of 5.5 months compared to 3.7 months for gemcitabine alone) and higher objective response rate.
	Hajj, et al. [30]	No	Yes	In vitro		Single-fraction 15 Gy	Combination produced significant growth delay compared to either TH-302 or XRT treatments alone.
**Clofibrate**	Xue, et al. [82]	No	Yes	In vivo		No chemotherapy Single-fraction 4 Gy	Reduction in the affinity of haemoglobin for oxygen and thus acting as a radiosensitiser for pancreatic xenografts.
**Papaverine**	Benej, et al. [25]	No	Yes	In vivo		No chemotherapy.	Significantly enhances tumour response to XRT in terms of LC and OS.
**PR-350**	Shibamoto, et al. [83]	No	Yes	In vitro and in vivo		Large amount required, therefore not predicted to have a high radiosensitising effect in clinical studies	Effective radiosensitiser in pancreatic cancer cell lines and xenografts.
	Sunamura, et al. [84]	No	Yes	Phase III clinical trial	48	Intraoperative XRT	PR-350 group showed higher survival rates and more effective control than the group that did not receive the radiosensitizer.
	Karasawa, et al. [85]	No	Yes	Phase III clinical trial	47	Intraoperative XRT No chemotherapy	No difference in short-term survival.
**Metformin**	Lipner, et al. [86]	No	No	In vitro		Monotherapy study.	All tested pancreatic cell lines were resistant to metformin.
	Benej, et al. [25]	No	Yes	In vivo		Metformin required 24 hours to reach full mitochondrial inhibition and clinical effectiveness	Papaverine was more suitable radiosensitiser, taking only 30 min to reach clinical effectiveness (similar to Atovaquone).
**OXY111A**	Limani, et al. [87]	No	No	Ib/IIa clinical trial (NCT02528526)	69	Study last updated at recruiting in 2015	Pending results. Study aims to assess the safety, tolerability, and efficacy of the HAP.
**PR-104**	Patterson, et al. [88]	Yes	Yes	In vitro		Single-fraction 10 Gy	Clinical benefit adding PR-104 to standard gemcitabine and XRT care.
	McKeage, et al. [89]	Yes		Phase Ib clinical trial (NCT00459836)	42	4 patients with pancreatic cancer, remaining 3 had other diseases	PR-104 combined with docetaxel results in dose-limiting toxicities.

HAP: hypoxia activated prodrug, PFS: progression-free survival, LC: local control, OS: overall survival, XRT: photon radiation therapy, Gy: Gray.

**Table 2:** Review of stereotactic body radiation therapy (SBRT) pancreatic cancer clinical studies (2000–current).

Author	Disease	Sample Size	Study Design	Chemotherapy	Total Dose (Gy) and Fractionation	Acute Side Effects Criteria for Adverse Events Version	Late Side Effects	MST Months (Range)	1-year OS Rate	2-year OS Rate	PFS	FFLP	Median FU Period Months (Range)
Chang, et al. [22] Φ *	Unresectable LAPC	77	Retrospective, single institute, combination of phase I and phase II studies.	Variety of gemcitabine-based regimens	25 in 1	Grade ≥ 2 = 5% NR	Grade ≥ 2 = 4% Grade ≥ 3 = 9%	11.8	21%	NR	1-year = 9%	1-year = 84%	6 (3–31)
Schellenberg, et al. [48] *	LAPC	20	Prospective, phase II trial, single institute.	Gemcitabine 1000 mg/m^2^ weekly (days 1, 8, and 15)	25 in 1	Grade ≥ 2 = 15% Grade 3 = 0% V3 [90]	Grade ≥ 2 = 15% Grade ≥ 3 = 5%	11.8	50%	20%	Median time to progression was 9.2 months	1-year = 94%	2 patients remaining alive = 25.1–36.4 months
Schellenberg, et al. [41] *	LAPC	16	Prospective, phase II, single institute.	Gemcitabine 1000 mg/m^2^ weekly (days 1, 8, and 15)	25 in 1	Grade 2 = 13% Grade 3 = 6% V3 [90]	Grade ≥ 2 = 33% Grade ≥ 3 = 13%	11.4	50%	Estimate 18%	Median time to progression was 9.7 months.	1-year = 100%	9.1 (22.3 for living patients)
Hoyer, et al. [47] *	Unresectable LAPC	22	Prospective, phase II, single institute.	NR	45 in 3	Grade ≥ 2 = 100% NR	Grade ≥ 2 = 94%	5.4	5%	NR	1-year = 9% Median time to progression was 4.8 months.	Local control rate = 57%	14 days–18 months
Wild, et al. [91] *	Recurrent	18	Reirradiation, retrospective, single institute.	5-fluorouracil-based regimen for 10 patients Gemcitabine- based regimen for 7 patients	25, 20 or 27 in 5 After chemoradiation of 50.4 Gy in 27	Grade 2 = 28% Grade 3 = 0% NR	Grade 3 = 6%	8.8	NR	NR	Median = 3.7 months	1-year = 62%	34.3 (6.4–61.6)
Macchia, et al. [92] *	Unresectable disease or recurrent	16	Prospective, phase I, single institute.	Variety of chemotherapy regimens	20–35 in 4–7	Grade 1 = 50% Grade 2 = 0% NR	Grade 3 = 6.3%	NR Overall response rate =56.2%	NR	50%	2-year distant progression free = 58.7%	2-year local progression free = 85.7%	24 (10–85)
Didolkar, et al. [39] *	Unresectable LAPC	85	Retrospective, single institute.	Variety of gemcitabine-based regimens post-SBRT	15–30 in 1–4	NR acute compared to late Grade ≥ 3 = 22.3% V2 [93]		18.6	50% Median 1-year = 13.4 months	NR	NR	Local control = 91.7%	NR (25.8 months at last follow up)
Mahadevan, et al. [54] †	LAPC	36	Retrospective, single institute.	Gemcitabine 1000 mg/m^2^ weekly (for 6 months)	24, 30 or 36 in 3	Grade 1 = 42% Grade 2 = 25% Grade 3 = 8% NR	Grade ≥ 3 = 6%	14.3	NR	NR	Median = 9.6 months	Local control = 78%	24 (12–33)
Mahadevan, et al. [3] †	LAPC	39	Retrospective, single institute.	Gemcitabine 1000 mg/m^2^ weekly (for 6 months)	24 or 30 in 3	Grade 1 = 41% Grade 2 = 23% Grade 3 = 0% V3 [90]	Grade 3 = 9%	20	NR	NR	Median = 15 months	Local control = 85% FFLP= 31%	21 (6–33)
Lominska, et al. [94] †	LAPC	28	Reirradiation, retrospective, single institute.	Variety of chemotherapy regimens	20–30 in 3–5 After 50.4 Gy XRT	Grade 2 = 4% V3 [90]	Grade 3 = 7%	5.9	18%	NR	NR	1-year = 70%	5.9 (1–27)
Dagoglu, et al. [95] †	Recurrent	30	Reirradiation, retrospective, single institute.	Variety of chemotherapy regimens Gemcitabine for 14 patients FOLFOX for 6 patients Erlotinib for 12 patients None for 5 patients	24–36 in 3–5	Grade 3 = 11% NR	Grade 3 = 7%	14	50%	5%	78%	NR	11 (4–24)
Tozzi, et al. [44] †	Unresectable LAPC = 21 Locally recurrent = 9	30	Prospective, single institute (consecutive enrolment).	Variety of gemcitabine-based regimens	36–45 in 6	Grade 1 = 43% Grade 2 = 10% Grade 3= 0% V3 [90]	Grade 3 = 0%	11	Median OS at 1-year = 47%	NR	Median PFS = 8 months	1-year = 96% (for 45 Gy group) and 85% for others	11.0 (2–28)
Gurka, et al. [96] †	LAPC (with elective nodes)	10	Prospective, single institute, pilot trial.	Concurrent gemcitabine with 1000 mg/m^2^ for 6 cycles	25 in 5	Grade 1 = 60% Grade 3 = 0% V3 [90]	Grade 2 = 0%	12.2	NR	NR	6.8 months	1-year = 40%	Until death
Koong, et al. [42] *	LAPC	15	Prospective, single institute, phase I.	Prior to enrolment 2 patients received conventional 5-FU–based chemoradiation to a dose of 50 Gy and 1 patient received chemotherapy alone.	15 (3 patients), 20 (5 patients), 25 (7 patients) in 1	Grade 1 = 13% Grade 2 = 20% Grade 3 = 0% GI toxicities were scored according to the Radiation Therapy Oncology Group acute radiation morbidity criteria.	NR	11	NR	NR	Median time to progression = 2 months	Local control = 75%	5
Koong, et al. [38] *	LAPC	16	Prospective, single institute, phase II.	Concurrent 5-fluorouracil	45 in 25 (IMRT) and 25 in 1 (SBRT)	Grade 0= 18.7% Grade 1= 43.7% Grade 2= 25% Grade 3= 12.5% GI toxicities were scored according to the Radiation Therapy Oncology Group acute radiation morbidity criteria.	NR	8.3	15%	NR	Median time to progression = 4.38 months	1-year = 8% Local control = 94%	5.75
Polistina, et al. [97] *	Unresectable LAPC	33	Prospective, single institute.	Gemcitabine 1000 mg/m^2^ weekly (for 6 weeks)	30 in 3	Grade 1 = 21.7% Grade 2 = 0% V3 [90]	NR	10.6	39.1%	0%	Median time to progression = 7.3 months	1-year = 82.6%	9
Rwigema, et al. [98] *	LAPC (mix of metastatic (11%), unresectable (56%) and recurrent disease (16%))	71	Retrospective, single institute.	Variety of chemotherapy regimens	18–25 in 1–3	Grade 1 = 24% Grade 2 = 11.3% Grade 3 = 4.2% NR	Grade 1 = 4.2%	10.3 months overall median OS	41%	NR	NR	Overall 1-year = 48.5% 1-year = 38% for unresectable 1-year = 18.8% for recurrent group 1-year = 40% for metastatic group	12.7 (4–26)
Herman, et al. [55] *	Unresectable LAPC	49	Prospective single-arm, multi-institutional, phase II.	Gemcitabine 1000 mg/m^2^ (3 doses) followed by a week break prior to SBRT	33 in 5	Grade ≥ 2 = 2% V4 [99]	Grade ≥ 2 = 11%	13.9 (10.2–16.7)	59%	18%	Median PFS = 7.8 months 1-year = 32% 2-year = 10%	1-year = 78%	13.9 (3.9–45.2)
Chuong, et al. [37] † (and *)	Nonmetastatic LAPC (16 patients) and borderline resectable pancreatic cancer (57))	73	Retrospective, single institute.	Induction gemcitabine-based regimens delivered over 3 cycles followed by SBRT	35–50 in 5	Grade ≥ 3 = 0 V4 [99]	Grade ≥ 3 = 5.3%	15 (LAPC) 16.4 (borderline)	68.1% (LAPC) 72.2% borderline	NR	Median PFS = 9.8 months 1-year PFS LAPC = 41% 1-year PFS borderline = 42.8%	1-year LC for non-surgical patients= 81%	10.5 (2.2–25.9)
Comito, et al. [40] *	Unresectable LAPC	45	Prospective, observational, single-arm, single institute, phase II.	71% completed regimens 2 weeks prior to SBRT 19% received gemcitabine-based regimens	45 in 6	Grade 1–2 = 49% Grade ≥ 3 = 0% V3 [90]	Grade 2 = 4% Grade ≥ 3 = 0%	19	85%	33%	Median PFS = 8 months	Median FFLP = 26 months 1-year = 87% 2-year = 87%	13.5 months (6–48)
Gurka, et al. [36] * (and †)	Borderline resectable and inoperable LAPC	38	Retrospective, single institute.	Variety of gemcitabine-based regimens	25–30 (one patient received 15) in 5	Grade 2 = NR Grade 3 = 5.2% V3 [90]	Grade 3 = 5.2% Grade 4 = 5.2% Grade 5 = 5.2%	14.3	NR	NR	9.2 months	Local control rate = 79%	NR
Mellon, et al. [46] *	Borderline resectable and LAPC	159 (110 BRPC and 49 LAPC)	Retrospective, single institute.	Variety of induction chemotherapy regimens	28–30 in 5	Grade1-2 = 52% Grade 3 = 11% V4 [99]	Grade 3 = 11%	19.2 (borderline) 15 (LAPC)	NR	NR	Event free survival = 11.9 months in borderline and 13.2 in LAPC	1-year locoregional control = 78%	5.6 (2.1–15.4)
Pollom, et al. [43] *	Unresectable (133), borderline resectable (11) pancreatic adenocarcinoma	167	Retrospective, single institute.	Variety of induction chemotherapy regimens (82% were gemcitabine-based)	25 in 1 (76 patients) 25–45 in 5 (91 patients)	Single-fraction: Grade ≥ 2 = 25% Multi-fraction: Grade ≥ 2 = 8.7% V4 [99]	Single-fraction: Grade ≥ 3 = 12.3% Multi-fraction: Grade ≥ 3 = 5.6%	13.6	Single-fraction= 30.8% Multi- fraction= 34.9%	NR	NR	NR	7.9 (0.1–63.6)

GI: gastrointestinal, MST: median survival time, OS: overall survival, XRT: photon radiation therapy, FU: follow up, NR: not reported, LAPC: locally advanced pancreatic cancer, IMRT: intensity modulated radiation therapy. Φ: Includes 40 patients from Schellenberg, et al. [41], Koong, et al. [42] and Koong, et al. [38]. *: OS measured from diagnosis †: OS measured from start of SBRT.

**Table 3:** Review of proton therapy pancreatic cancer clinical studies (2000–current).

Author	Disease	Sample Size	Study Design	Chemotherapy	Total Dose and Fractionation	Acute Side Effects Criteria for Adverse Events Version	Late Side Effects	MST Months (Range)	1-year OS Rate	2-year OS Rate	PFS	FFLP	Median FU Period Months (Range)
Hiroshima, et al. [45] †	Unresectable LAPC	42	Retrospective, single institute.	Concurrent chemotherapy (gemcitabine (38 patients) or S-1 (3 patients)).	50 GyE (12 patients) and 54–67.5 GyE (30 patients) in 25-33	Grade 1 = 9.5% Grade 2 = 36% Grade 3 = 40% Grade 4 = 4.8% V4 [99]	Grade 1 = 7% Grade 2 = 4.8% Grade 3 = 0%	25.6	77.8%	50.8%	Median time to local recurrence = 36 months	1-year LC rate = 83.3% 2-year LC rate = 78.9%	14 (2.4–47.6)
Murphy, et al. [100] †	Borderline resectable PDAC	48	Prospective, single institute, phase II trial.	Neoadjuvant FOLFIRINOX (8 cycles). Vascular involvement resolution determined whether patients received short-course capecitabine or long-course fluorouracil or capecitabine chemoradiation.	25 GyE in 5	Grade 3 GI = 10% V4 [99]	NR	37.7	NR	56%	14.7 months 2-year PFS = 43	NR	18
Sachsman, et al. [50] Φ	Unresectable LAPC	11	Prospective, single institute, phase II trial.	Concomitant capecitabine (1000 mg orally twice daily) 5 days/week	59.4 Gy (RBE) in 33	Grade 2 = 9% Grade 3 = 0% NR	Grade 2 = 0% Grade 3 = 0%	18.4	61%	31%	1-year =55% 2-year =14%	1-year = 86% 2-year = 69%	14 (5–25) For surviving patients 23 (8–25)
Terashima, et al. [52] §	LAPC (T3-T4) regardless of adjacency	40	Prospective, single institute, phase I/II trial.	Concurrent gemcitabine 800 mg/m^2^ weekly, for 30 min for the initial 3 weeks (days 1, 8, and 15) during 5 weeks of PT	67.5 GyE in 25	Grade 3 = 95% Grade 4 = 7.5% V3 [90]	Grade 3 = 8% Grade 5 = 2%	NR	78.8%	NR	1-year = 60.8%	1-year = 79.9%	12.1 (3.2–22.3)
Terashima, et al. [52] §	LAPC (T3-T4) adjacent to the GI	5	Prospective, single institute, phase I/II trial.	Concurrent gemcitabine 800 mg/m^2^ weekly, for 30 min for the initial 3 weeks (days 1, 8, and 15) during 5 weeks of PT	50 GyE in 25	Grade 3 = 100% V3 [90]	NR	NR	NR	NR	NR	NR	12.3 (8.2–18.6)
Terashima, et al. [52] §	LAPC (T3-T4) non-adjacent to the GI	5	Prospective, single institute, phase I/II trial.	Concurrent gemcitabine 800 mg/m^2^ weekly, for 30 min for the initial 3 weeks (days 1, 8, and 15) during 5 weeks of PT	70.2 GyE in 26	Grade 3 = 100% V3 [90]	Grade 3 = 100%	NR	NR	NR	NR	NR	19.6 (17.7–21.5)
Terashima, et al. [52]	Combined group	50	Prospective, single institute, phase I/II trial.	Concurrent gemcitabine 800 mg/m^2^ weekly, for 30 min for the initial 3 weeks (days 1, 8, and 15) during 5 weeks of PT	50–70.2 GyE in 25–26			NR	76.8%	NR	64.3%	1-year = 81.7%	12.5
Nichols, et al. [59]	Pancreatic and ampullary adenocarcinoma Resected = 5, marginally resectable = 5, and unresectable = 12.	22	Prospective, single institute.	Concomitant capecitabine (1000 mg orally twice daily)	50.4–59.4 CGE in 28–33	Grade 2 = 13.6% Grade 3 = 0% V4 [99]	NR	11= resected 14 = for marginally resectable 8.8 = unresectable	NR	NR	NR	NR	11 (5–36)
Hitchcock, et al. [60]	Unresectable LAPC	15	Retrospective, single institute.	Concomitant capecitabine (1000 mg orally twice daily)	59.40 Gy (RBE) in 33 50.40 Gy (RBE) for 1 patient	NR	NR	24 (10–30) for 5 resected patients	NR	NR	NR	NR	NR
Takatori, et al. [53] *	Unresectable LAPC	91	Prospective, single institute.	Concurrent gemcitabine (800 mg/m^2^ on days 1, 8, and 15) for the initial 3 weeks during 5 weeks of PT	67.5 GyE in 25	NR Gastric/duodenal ulcers incidence = 49.4% V3 [90]	Grade 4 GI = 1% Grade 5 GI = 2%	NR	NR	NR	NR	NR	10
Hong, et al. [62] ^	Resectable PDAC	3	Prospective, single institute, phase I trial.	Concurrent capecitabine at 825 mg/m^2^ orally twice daily	25 Gy (RBE) in 5	Grade 3 = 67% NR	NR	NR	NR	NR	NR	NR	12
Hong, et al. [62] ^	Resectable PDAC	12	Prospective, single institute, phase I trial.	Concurrent capecitabine at 825 mg/m^2^ orally twice daily	30 Gy (RBE) in 10	Grade 3 = 16% NR	NR	NR	NR	NR	NR	NR	12
Combined Hong, et al. [62]	Resectable PDAC	15	Prospective, single institute, phase I trial.	Concurrent capecitabine at 825 mg/m^2^ orally twice daily	25–30 Gy (RBE) in 5–10	Grade 3 = 27% NR	NR	NR	75%	NR	Median relapse free survival was 10 months	NR	12
Hong, et al. [63]	Resectable PDAC	50	Prospective, single institute phase I/II study. 15 patients from Hong, et al. [62] phase I study.	Concurrent capecitabine at 825 mg/m^2^ orally twice daily. Gemcitabine for 6 months starting post-operative 4 to 10 weeks	25 GyE in 5	Grade 2 = 31.4% Grade 3 = 4.1% (phase II patients only) Grade 4 = 0% NR	NR	17.3 (11.2–29.2) months for non-resected For the 37 eligible resected patient = 27.0 (16.2–32.3)	NR	42%	10.4 (7.5–17.1) months for non-resected For the 37 Eligible resected patient = 14.5 (10.2–21.8) Whole group = 10	Locoregional failure occurred = 16.2% Distant recurrence occurred = 72.9%	12 patients alive at 38 months
Boimel, et al. [101]	Locally recurrent LAPC	15	Reirradiation study, retrospective, single institute.	Variety of chemotherapy regimens. 67% of patients received concurrent chemotherapy	37.5–59.4 Gy (RBE) Prior radiation dose 30–59.4 Gy	Grade ≥ 3 = 13% V4 [99]	NR	16.7	67%	NR	Distant metastasis free survival 1-year = 64%	72%	15.7 (2–48)
Jethwa, et al. [58] £	LAPC	13	Retrospective, non-randomised, single institute.	Concurrent capecitabine 825mg/m^2^ twice daily. 2 patients received concurrent 5-fluorouracil 225mg/m^2^	50 Gy (RBE) in 25	Grade 1 = 46% Grade 2 = 15% Grade ≥ 3 = 0% V4 [99]	NR	NR	62%	40%	1-year local control rate = 66%	1- and 2- year freedom from distant metastasis rate = 53% and 28%	16 (9–24)
Kim, et al. [57]	LAPC (4 recurrent, 1 metastatic)	37	Retrospective, non-randomised, single institute.	Variety of chemotherapy regimens. 21.6% patients received induction chemotherapy	45 and 30 GyE in 10	Anaemia: Grade 1 = 32.4% Grade 2 = 8.1% Leukopenia: Grade 1 = 21.4% Grade 2 = 2.7% Grade 1 thrombocytopenia= 2.7% Grade 1 abdominal pain = 16.2% Anorexia: Grade 1 = 10.8% Grade 2 = 8.1% Stomatitis: Grade 1 = 2.7% Grade 2 = 2.7% Vomiting: Grade 1 = 8.1% Grade 2 = 5.4% Grade ≥ 3 = 0% V4 [99]	Grade 1-2 = NR Grade ≥ 3 = 0%	19.3	OS rates = 75.7%	NR	Relapse free survival = 33.2%	64.8%	16.7 months (2.3–32.1 months)
Maemura, et al. [71]	Unresectable LAPC	25	Prospective, non-randomised, single institute.	Induction and concurrent chemotherapy (gemcitabine or S-1)	50 Gy (XRT) or 67.5 GyE (PT) in 25	XRT = higher incidence of haematological toxicity, grade 3 = 3 patients PT = grade 2 or 3 gastric ulcer = 2 patients V4 [99]	NR	XRT = 23.4 PT = 22.3	XRT = 86.7% PT = 80%	XRT = 33.3% PT = 45%	Median time (15.4 months) to progression was the same across both PT and XRT.	Local progression: XRT = 40% PT = 60% Disease control rates: XRT = 93% PT = 80%	NR
Tseng, et al. [64] ‡ *	Resectable LAPC	47	Retrospective, single institute.	Concurrent neoadjuvant capecitabine (825 mg/m^2^ twice daily over 1 week (41 patients) or 2 weeks (6 patients) for 5 days a week	25 GyE in 5	Grade 1 = 51% Grade 2 = 4% Grade ≥ 3 = 0% V3 [90]	NR	NR	NR	NR	NR	NR	8.5 (7 days–18.6 months)

RBE: relative biological effectiveness, GI: gastrointestinal, MST: median survival time, OS: overall survival, XRT: photon radiation therapy, PT: proton therapy, FU: follow up, NR: not reported, LAPC: locally advanced pancreatic cancer, PFS: progression-free survival, PDAC: pancreatic ductal adenocarcinoma. Φ: OS measured from start of treatment. *: follow up from the end of radiation. †: OS measured from start of chemoradiation. §: Terashima, et al. [52] reported on 3 dosing protocols based on disease. Separate results are reported where available, ‡: Patient overlap with Hong, et al. [62]. ^: Hong, et al. [62] reported on 2 dosing levels. Separate results are reported where available. £: Jethwa, et al. [58] reported on 2 chemotherapy regimens. Separate results are reported where available. Dose to target volumes: RBE: Relative biological effective dose. CGE: Cobalt Gray equivalents (absorbed dose × 1.1 (RBE) to express the biologic effective proton dose). GyE: Gray equivalents (proton physical dose (in Gray) × 1.1 (RBE)).

**Table 4:** Review of carbon ion therapy pancreatic cancer clinical studies (2000–current).

Author	Disease	Sample Size	Study Design	Chemotherapy	Total Dose and Fractionation	Acute Side Effects Criteria for Adverse Events Version	Late Side Effects	MST Months	1-year OS Rate	2-year OS Rate	PFS	FFLP	Median FU Period Months (Range)
Shinoto, et al. [68] Φ	Unresectable LAPC	64	Retrospective, single institute.	Gemcitabine or S-1	55.2 Gy (RBE) in 12	Grade 2 = 26% Grade 3 = 6% V4 [99]	Grade 2 = 6% Grade ≥3 = 0%	25.1	84%	53%	2-year = 23%	2-year LC = 82%	24.4 (5.1–46.1)
Kawashiro, et al. [17] Φ	Unresectable LAPC	72	Retrospective, non-randomized, multi- institutional study.	68% received concurrent gemcitabine (1000 mg/m^2^ weekly)	52.8 Gy (RBE) or 55.2 Gy (RBE) in 12	Grade 2 = 44% Grade 3 = 28.1% Grade 4 = 1% V4 [99]	Grade 1 = 99% Grade 2 = 0% Grade 3 = 1%	21.5	73%	46%	Local recurrence incidence at 1- year and 2- year = 16% and 24%	Distant metastasis-free survival at 1-year and 2-year = 41% and 28%. Median distant metastasis-free survival = 8.3 months	13.6 (2.8–37.9) For surviving patients 14.7 (3.2–37.5)
Combs, et al. [102]	LAPC	33	Prospective, phase I, single institute.	Concurrent gemcitabine (300 mg/m^2^)	45–53 GyE in 3	PHOENIX-01 trial withdrawn (before enrolment).							
Shinoto, et al. [67] Φ	Unresectable LAPC	72	Prospective, single institute.	Concurrent gemcitabine (400–100 mg/m^2^) on days 1,8 and 15.	43.2–55.2 GyE in 12	Grade 1 ≥ GI ulcer = 15% Grade ≥ 3 haematologic toxicities = 53% V3 [90]	Grade 3 = 1.4%	19.6	73%	35% overall For ≥ 45.6 GyE group 2-year OS = 48%	The median time to progression was 5.9 months. 86% experienced distant metastases	1-year = 92% 2-year = 83%	≥ 2 years
Shinoto, et al. [70] Φ	Potentially resectable LAPC	26	Phase 1, single institute.	NR	30–36.8 GyE in 8	Grade 1 = 3.8% Grade 3 = 3.8% V2 [93]	Grade 4 = 3.8%	18.6	69% For patient who underwent surgical resection = 81%	NR	No patients experienced local recurrence. distant metastasis in 65% of patients	81% of patients underwent surgery. 5-year survival rates for all 26 patients and for those who underwent surgery were 42% and 52%	33.8
Shinoto, et al. [103]	LAPC	45	Phase II, single institute.	Concurrent S-1 administered orally twice a day (80 mg/m^2^) for 28 days every 6 weeks.	55.2 GyE in 12	Currently recruiting							

RBE: relative biological effectiveness, GI: gastrointestinal, MST: median survival time, OS: overall survival, RT: radiation therapy, FU: follow up, NR: not reported, LAPC: locally advanced pancreatic cancer, PFS: Progression-free survival. Φ: OS measured from start of treatment. Dose to target volumes: RBE: relative biological effective dose. GyE: Gray equivalents (carbon physical dose (in Gray) × (RBE)).

## Data Availability

Data supporting the results reported in the article can be found on the University of South Australia database and are available upon request.

## Appendix A

**Table:**

	Search Term	Hits
1	(proton therap * or proton beam * or proton pencil beam *).mp.	6280
2	(carbon ion therap * or carbon beam *).mp.	425
3	1 or 2	6594
4	(Radiol * or radiotherapy * or radiation therap * or chemoradio * or Radiosurger * or stereotactic * or SBRT or SABR or ablative * or dose escalat * or dose-escalat *).mp.	719,296
5	exp Radiotherapy	177,645
6	4 or 5	732,871
7	3 and 6	5310
8	((vasodilator * or oxygenat * or vessel * or dilat * or oxygen * or vasostimula * or hypox * or oxygen-mimetic or hypoxia-activated) adj3 (radiosensiti* or activated? or prodrug * or agent * or stimulated * or therapy? or drug * or pharmaceutical? or drug * or HAP)).mp.	80,168
9	exp Pancreatic Neoplasms	72,324
10	((Pancreas or pancreatic or LAPC or PDAC or ductal) adj3 (cancer * or neoplasm * or malignan * or tumor? * or carcinoma ? or adenocarcinoma ?)).mp.	117,214
11	9 or 10	119,161
12	6 and 8 and 11	32
13	7 and 8 and 11	1
14	12 or 13	32
15	7 or 11	70
16	14 or 15	101
17	Limit 16 to English language	95
18	Limit 17 to yr = “2000–Current”	87

*: symbol used to include a variation of word endings.

## Appendix B

Exclusion criteria:

Conference abstracts
Case studies
Dosimetric studies
3D conformal radiation therapy treatment
